# Autoantibodies to N-terminally truncated GAD improve clinical phenotyping of individuals with adult-onset diabetes: Action LADA 12

**DOI:** 10.1007/s00125-018-4605-3

**Published:** 2018-04-04

**Authors:** Peter Achenbach, Mohammed I. Hawa, Stephanie Krause, Vito Lampasona, Samuel T. Jerram, Alistair J. K. Williams, Ezio Bonifacio, Anette G. Ziegler, R. David Leslie, on behalf of the Action LADA consortium

**Affiliations:** 1 Institute of Diabetes Research, Helmholtz Zentrum München, German Research Center for Environmental Health, Ingolstaedter Landstrasse 1, 85764 Munich-Neuherberg, Germany; 2 Forschergruppe Diabetes, Technische Universität München, at Klinikum rechts der Isar, Munich, Germany; 3 German Center for Diabetes Research (DZD), Munich-Neuherberg, Germany; 4 Forschergruppe Diabetes e.V. at Helmholtz Zentrum München, German Research Center for Environmental Health, Munich-Neuherberg, Germany; 5 Centre for Immunobiology, Blizard Institute, Barts and the London School of Medicine and Dentistry, Queen Mary University of London, London, E1 2AT UK; 6 Division of Genetics and Cell Biology, San Raffaele Scientific Institute, Milan, Italy; 7 Diabetes and Metabolism, School of Clinical Sciences, Southmead Hospital, University of Bristol, Bristol, UK; 8 DFG Research Center for Regenerative Therapies Dresden, Faculty of Medicine, Technische Universität Dresden, Dresden, Germany; 9 Paul Langerhans Institute Dresden, German Center for Diabetes Research (DZD), Technische Universität Dresden, Dresden, Germany

**Keywords:** Adult-onset diabetes, Autoantibodies, Autoimmune, Clinical phenotype, GAD, LADA, N-terminally truncated GAD65, Patients, Type 1 diabetes

## Abstract

**Aims/hypothesis:**

Adult-onset type 1 diabetes, in which the 65 kDa isoform of GAD (GAD65) is a major autoantigen, has a broad clinical phenotype encompassing variable need for insulin therapy. This study aimed to evaluate whether autoantibodies against N-terminally truncated GAD65 more closely defined a type 1 diabetes phenotype associated with insulin therapy.

**Methods:**

Of 1114 participants with adult-onset diabetes from the Action LADA (latent autoimmune diabetes in adults) study with sufficient sera, we selected those designated type 1 (*n* = 511) or type 2 diabetes (*n* = 603) and retested the samples in radiobinding assays for human full-length GAD65 autoantibodies (f-GADA) and N-terminally truncated (amino acids 96–585) GAD65 autoantibodies (t-GADA). Individuals’ clinical phenotypes were analysed according to antibody binding patterns.

**Results:**

Overall, 478 individuals were f-GADA-positive, 431 were t-GADA-positive and 628 were negative in both assays. Risk of insulin treatment was augmented in t-GADA-positive individuals (OR 4.69 [95% CI 3.57, 6.17]) compared with f-GADA-positive individuals (OR 3.86 [95% CI 2.95, 5.06]), irrespective of diabetes duration. Of 55 individuals who were f-GADA-positive but t-GADA-negative, i.e. with antibody binding restricted to the N-terminus of GAD65, the phenotype was similar to type 2 diabetes with low risk of progression to insulin treatment. Compared with these individuals with N-terminal GAD65-restricted GADA, t-GADA-positive individuals were younger at diagnosis (*p* = 0.005), leaner (*p* < 0.0001) and more often had multiple diabetes-associated autoantibodies (28.3% vs 7.3%; *p* = 0.0005).

**Conclusions/interpretation:**

In individuals with adult-onset diabetes, presence of N-terminally truncated GAD65 autoantibodies is associated with the clinical phenotype of autoimmune type 1 diabetes and predicts insulin therapy.

**Electronic supplementary material:**

The online version of this article (10.1007/s00125-018-4605-3) contains peer-reviewed but unedited supplementary material, which is available to authorised users.

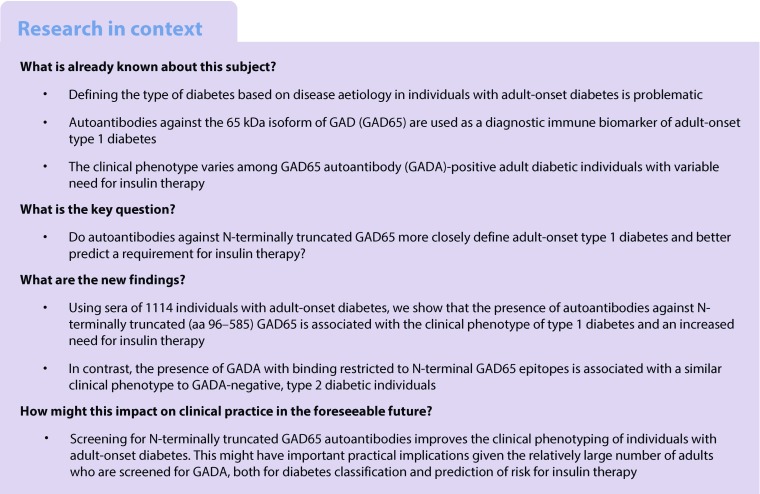



## Introduction

Type 1 diabetes can occur at any age and is of variable severity. In adults presenting with the disease, unlike children, it may masquerade as type 2 diabetes, since insulin therapy is not always required [1]. Established autoimmune markers of type 1 diabetes, notably GAD autoantibodies (GADA), have been used to screen individuals presenting with adult-onset diabetes to identify those with type 1 diabetes, including the so-called latent autoimmune diabetes in adults (LADA) [2, 3]. Identifying individuals with adult-onset type 1 diabetes is important as, compared with people with type 2 diabetes, they have a different clinical course and different comorbidities [1]. In adult-onset diabetes, the detection of GADA together with insulinoma-associated antigen-2 autoantibodies (IA-2A) or zinc transporter-8 autoantibodies (ZnT8A) is highly predictive of the need for insulin therapy [4]. Nevertheless, the majority of antibody-positive individuals with adult-onset diabetes present with GADA alone, which may represent either false-positive antibody assay signals in an adult population with a relatively low prevalence of autoimmune type 1 diabetes, or true-positive autoantibodies with high risk for progression to insulin therapy. Antibody characteristics might facilitate diagnosis and estimation of risk for disease progression because high-affinity GADA responses against the middle (amino acids [aa] 235–444) and C-terminal (aa 445–585) domains of the 65 kDa isoform of GAD (GAD65) were associated with type 1 diabetes development in children and adolescents [5]; and high antibody titre [6] and binding to C-terminal (aa 437-585) GAD65-depending epitopes were reported as characteristic features of LADA individuals [7]. In contrast, low-affinity GADA and/or antibody responses restricted to the N-terminal (aa 1–95) domain of GAD65 were not associated with classic type 1 diabetes [5, 8]. Furthermore, using N-terminally truncated (aa 96–585) GAD65 antigen in GADA radiobinding assays improved the specificity for type 1 diabetes [9]. The aim of this study was to evaluate N-terminally truncated (aa 96–585) GAD65 autoantibodies (t-GADA), compared with full-length GAD65 autoantibodies (f-GADA), in a large cohort of adult-onset diabetes individuals with variable clinical phenotype.

## Methods

### Individuals and samples

Sera were obtained from individuals with adult-onset diabetes participating in the Action LADA study [3]. The study design is cross-sectional and includes individuals with adult-onset diabetes (originally *n* = 6156; aged 30–70 years; diabetes diagnosed within 5 years) with the aim of identifying risk factors for adult-onset autoimmune diabetes (www.actionlada.org). The study protocol is in accordance with the Declaration of Helsinki and was approved by local ethics committees. Informed written consent was obtained from all individuals before blood sampling. The study was approved by the UK National Research Ethics Committee (no. P/02/240).

All samples had previously been tested for GADA, IA-2A and ZnT8A in the central laboratory in London [3]. For the current study, we selected two groups from our initial cohort: of those with sufficient sera in storage, 511 individuals with beta cell autoantibodies (all GADA-positive), and 603 individuals without beta cell autoantibodies of similar age and diabetes duration (Table 1). Of these 1114 participants, the median age at diagnosis was 48.2 years (interquartile range [IQR] 39.5–56.3 years) and median disease duration at sample collection was 2.0 years (IQR 0.6–3.5 years); while information on diabetes treatment and BMI was available for 1045 (94%) and 1059 (95%) individuals, respectively.

**Table 1:** Randomly selected beta cell autoantibody positive and negative individuals from the Action LADA cohort (*n* = 1114), grouped according to binding patterns of f-GADA and t-GADA. Data are presented as median (IQR) or *n* (%)

	Beta cell autoantibody positive	Beta cell autoantibody negative	f-GADA negative, t-GADA negative	f-GADA positive, t-GADA negative (restricted)	f-GADA positive	t-GADA positive	f-GADA positive, t-GADA positive
Samples, *n*	511	603	628	55	478	431	423
Female, *n* (%)^a^	264 (51.9)	281 (46.6)	289 (46.0)	24 (43.6)	251 (52.7)	232 (54.1)	227 (53.9)
Male, *n* (%)^a^	245 (48.1)	322 (53.4)	339 (54.0)	31 (56.4)	225 (47.3)	197 (45.9)	194 (46.1)
Age (years)	49.3 (40.7–58.2)	50.8 (41.7–58.6)	51.2 (41.9–58.7)	54.2 (43.6–62.5)	49.0 (40.2–57.6)	48.2 (39.8–57.4)	48.2 (39.7–57.2)
Age at onset (years)	47.6 (38.6–56.0)	48.9 (40.3–56.6)	48.9 (40.5–56.7)	52.0 (41.8–59.8)	47.0 (38.4–55.7)	46.6 (38.0–55.5)	46.4 (37.9–55.1)
Duration of disease (years)	2.0 (0.8–3.6)	1.8 (0.5–3.5)	2.0 (0.5–3.5)	1.8 (0.3–3.10)	2.0 (0.7–3.5)	2.0 (0.8–3.5)	2.0 (0.8–3.5)
BMI (kg/m^2^)^b^	26.0 (22.9–30.1)	29.5 (26.6–33.8)	29.7 (26.6–34.1)	29.9 (26.7–35.9)	25.6 (22.8–29.4)	24.9 (22.6–28.8)	24.8 (22.6–28.8)
Individuals on insulin, *n* (%)^c^	238 of 487 (48.9)	120 of 558 (21.5)	126 of 587 (21.5)	7 of 51 (13.7)	231 of 451 (51.2)	225 of 407 (55.3)	224 of 400 (56.0)

Missing information for ^a^sex (*n* = 2), ^b^BMI (*n* = 55), ^c^diabetes treatment (*n* = 69)

### Antibody measurements

Sera of the 1114 participants were retested for GADA in the Munich laboratory using the National Institute of Diabetes and Digestive and Kidney diseases (NIDDK) harmonised radiobinding assay [10]. In vitro transcribed/translated and [^35^S]-labelled human full-length (aa 1–585) GAD65 and N-terminally truncated (aa 96–585) GAD65 (cloned by Vito Lampasona, San Raffaele Institute, Milan, Italy) were used as antigens to detect f-GADA and t-GADA, respectively. The assays achieved adjusted sensitivities of 64% (f-GADA) and 72% (t-GADA) at 95% specificity in the 2012 Islet Autoantibody Standardization Program workshop [9]. Throughout all measurements, investigators were blinded with respect to the previously determined autoantibody status of the samples.

### Statistical analysis

We used the Mann–Whitney *U* test to compare continuous variables, Fisher’s exact test to compare frequencies and Spearman correlation to determine the correlation between antibody titres. Data are presented, where appropriate, as medians with IQRs or ORs with 95% CIs. For all analyses, a two-tailed *p* value of <0.05 was considered significant. Statistical analyses were performed using the GraphPad Prism 3 program (GraphPad Software, La Jolla, CA, USA).

## Results

**Fig. 1:**
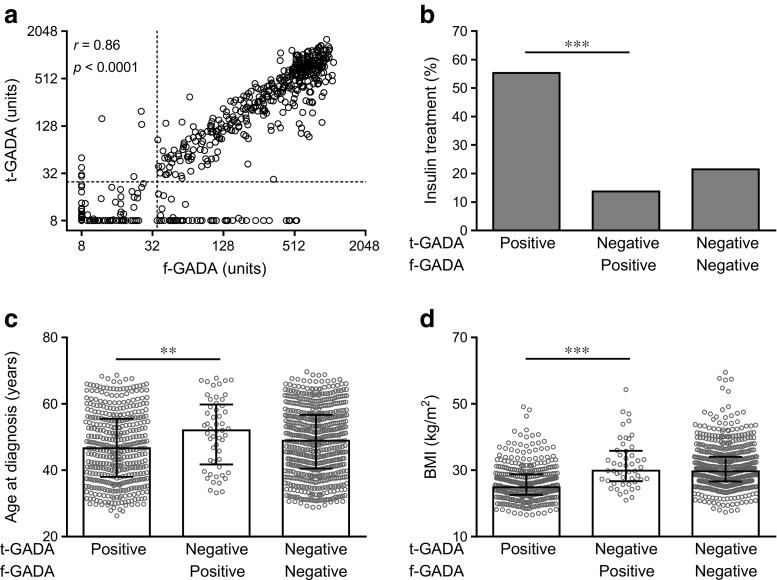
Relationship between GADA epitope reactivity and clinical phenotype. In (**a**), levels of f-GADA (*x*-axis) are plotted against levels of t-GADA (*y*-axis) using log_2_ scales for 1114 participants with adult-onset diabetes. Dashed lines indicate thresholds for positivity for f-GADA (35 units) and t-GADA (25 units). In (**b–d**), individuals are grouped according to positive or negative status of f-GADA and t-GADA, and compared with respect to frequency of insulin treatment (**b**), age at diagnosis (**c**) and BMI (**d**). Median (IQR) values are shown for each group in (**c**) and (**d**). **p < 0.01, ***p < 0.001

Of 1114 participants with adult-onset diabetes, 478 were f-GADA-positive using the NIDDK harmonised radiobinding assay. Of these 478 f-GADA-positive individuals, 55 (11.5%) were t-GADA-negative, thus demonstrating antibody binding restricted to N-terminal GAD65 epitopes (Fig. 1a). Such restricted responses were seen in four (3.2%) of the 126 f-GADA-positive individuals who also had IA-2A and/or ZnT8A (electronic supplementary material [ESM] Fig. 1a), in contrast to 51 (14.5%) of the 352 individuals who only had f-GADA positivity (ESM Fig. 1b; *p* = 0.0003). Overall, 431 individuals were t-GADA-positive. Antibody titres were strongly correlated between f-GADA and t-GADA assays (*r* = 0.86, *p* < 0.0001).

For clinical phenotype, f-GADA-positive individuals were diagnosed with diabetes at a median age of 47.0 years (IQR 38.4–55.7 years), median BMI of 25.6 kg/m^2^ (IQR 22.8–29.4 kg/m^2^) and 51.2% required insulin therapy (Table 1). For the t-GADA-positive individuals, median age at diagnosis was 46.6 years (IQR 38.0–55.5 years), median BMI 24.9 kg/m^2^ (IQR 22.6–28.8 kg/m^2^) and 55.3% were on insulin therapy. For the whole cohort, risk of insulin treatment was augmented in t-GADA-positive individuals (OR 4.69 [95% CI 3.57, 6.17]; *p* < 0.0001 vs t-GADA-negative) compared with f-GADA-positive individuals (OR 3.86 [95% CI 2.95, 5.06]; *p* < 0.0001 vs f-GADA-negative). This difference was evident irrespective of the disease duration: both for the cohorts assessed at 1 month post-diagnosis (OR 5.20 [95% CI 3.89, 6.94] and 4.42 [3.32, 5.88], respectively) and at 6 months post-diagnosis (OR 4.92 [95% CI 3.61, 6.69] and 4.25 [95% CI 3.13, 5.77], respectively).

Strikingly, when comparing the t-GADA-positive individuals with individuals who were N-terminal GAD65-restricted in antibody binding, i.e. f-GADA-positive but t-GADA-negative, the latter required insulin treatment less often (13.7%; *p* < 0.0001; Fig. 1b), were older at diagnosis (median age 52.0 years [IQR 41.8–59.8 years]; *p* = 0.005; Fig. 1c) and were more obese (median BMI 29.9 kg/m^2^ [IQR 26.7–35.9 kg/m^2^]; *p* < 0.0001; Fig. 1d). Moreover, individuals with N-terminal GAD65-restricted antibodies were not phenotypically different from individuals who were negative for both f-GADA and t-GADA, i.e. type 2 diabetes individuals (Fig. 1, Table 1).

Finally, when comparing the individuals who were positive (*n* = 423) or negative (*n* = 628) in both assays (f-GADA and t-GADA), these positive individuals were younger at diagnosis (*p* = 0.003), leaner (*p* < 0.0001) and were more often on insulin treatment (*p* < 0.0001) (Table 1).

## Discussion

In this study, we found that overall, f-GADA-positive participants and t-GADA-positive participants with adult-onset diabetes had a similar clinical phenotype that distinguished them from GADA-negative participants with type 2 diabetes. Importantly, however, a subgroup of individuals who were positive for f-GADA but negative for t-GADA (i.e. individuals with N-terminal GAD65-restricted GADA) had a similar clinical phenotype to GADA-negative, type 2 diabetic individuals, albeit in relatively low numbers. It is therefore possible that the N-terminal GAD65-restricted GADA, as proposed, does not detect individuals with autoimmune type 1 diabetes, at least using clinical phenotype as a proxy, whilst screening for t-GADA could be helpful for clinical assessment and prediction of insulin therapy.

Screening adult-onset diabetes individuals for GADA to identify autoimmune type 1 diabetes is limited by false-positive and false-negative assay results [1]. Using the t-GADA assay, when compared with the f-GADA assay, we identified a reduced number of GADA-positive individuals (88%) and confirmed a high number (99%) of GADA-negative individuals. Further, we found that the risk of being on insulin treatment within 5 years from diagnosis when positive for t-GADA was higher (OR 4.69) than in those individuals positive for f-GADA (OR 3.86). Those positive for t-GADA, compared with those with N-terminal GAD65-restricted GADA, were enriched for phenotypic features characteristic of individuals with adult-onset type 1 diabetes, including being younger, leaner and with an increased need for insulin treatment.

Differences in GADA epitope binding between LADA individuals with clinical characteristics similar to either type 1 or type 2 diabetes have previously been reported [7]. Individuals with antibodies against epitopes within the N-terminal/middle (aa 1–360) GAD65 region and negative for antibodies against the C-terminal (aa 437–585) GAD65 region showed clinical features that were indistinguishable from those of GADA-negative individuals, whereas the presence of autoantibodies directed to both GAD65 regions identified a subgroup of individuals with low BMI, low basal C-peptide values and the need for insulin treatment [7]. In contrast to this former study and based on our previous data [5, 9], we used the N-terminally truncated (aa 96–585) GAD65 antigen for our t-GADA assay, containing epitopes within the middle and C-terminal GAD65 regions. We believe that the false-positive rate in screening tests can likely be decreased by using this t-GADA assay to identify those at risk of progression to insulin treatment.

In conclusion, our findings suggest that in individuals with adult-onset diabetes, screening for t-GADA augments the clinical phenotype of autoimmune type 1 diabetes. This may have important practical implications given the relatively large number of adult patients who are screened for GADA, both for diabetes classification and prediction of risk for insulin therapy.

### Electronic supplementary material

ESM Figure 1 (PDF 89 kb)

## Data Availability

The datasets generated and/or analysed during the current study are available from the corresponding author on reasonable request.

## Appendix: Members of the Action LADA consortium

Richard David Leslie and Mohammed I Hawa, Blizard Institute, Queen Mary University of London, London, UK

Paolo Pozzilli, University Campus Bio-Medico, Rome, Italy

Henning Beck-Nielsen and Knud Yderstraede, University Hospital of Odense, Odense, Denmark

Steven Hunter and David Hadden, Royal Victoria Hospital, Belfast, UK

Raffaella Buzzetti, University La Sapienza, University of Rome, Rome, Italy

Werner Scherbaum and Hubert Kolb, University of Düsseldorf, Düsseldorf, Germany

Nanette C. Schloot, German Diabetes Centre, University of Düsseldorf, Düsseldorf and Clinic for Metabolic Diseases at University Hospital Düsseldorf, Germany. N.C. Schloot is guest scientist at the German Diabetes Center, Institute of Clinical Diabetology, Düsseldorf and currently employed by Lilly Deutschland, Bad Homburg, Germany

Jochen Seissler, Ludwig-Maximilians-University, Munich, Germany

Guntram Schernthaner, Rudolfstiftung Hospital, Vienna, Austria

Jaakko Tuomilehto and Cinzia Sarti, National Institute for Health and Welfare, Helsinki, Finland

Alberto De Leiva and Eulalia Brugues, Universitat Autonoma de Barcelona, Barcelona, Spain

Didac Mauricio, University Hospital Germans Trias Pujol, Badalona, Spain

Charles Thivolet, Hopital Edouard Herriot, Lyon, France

## Abbreviations

aa: Amino acids
GAD65: 65 kDa isoform of GAD
GADA: GAD autoantibodies
f-GADA: Full-length (aa 1–585) GAD65 autoantibodies
t-GADA: N-terminally truncated (aa 96–585) GAD65 autoantibodies
IA-2A: Insulinoma-associated antigen-2 autoantibodies
IQR: Interquartile range
LADA: Latent autoimmune diabetes in adults
NIDDK: National Institute of Diabetes and Digestive and Kidney diseases
ZnT8A: Zinc transporter-8 autoantibodies

## References

[1] Leslie RD, Palmer J, Schloot NC, Lernmark A (2016). Diabetes at the crossroads: relevance of disease classification to pathophysiology and treatment. Diabetologia.

[2] Tuomi T, Groop LC, Zimmet PZ, Rowley MJ, Knowles W, Mackay IR (1993). Antibodies to glutamic acid decarboxylase reveal latent autoimmune diabetes mellitus in adults with a non-insulin-dependent onset of disease. Diabetes.

[3] Hawa MI, Kolb H, Schloot N (2013). Adult-onset autoimmune diabetes in Europe is prevalent with a broad clinical phenotype: Action LADA 7. Diabetes Care.

[4] Lampasona V, Petrone A, Tiberti C (2010). Zinc transporter 8 antibodies complement GAD and IA-2 antibodies in the identification and characterization of adult-onset autoimmune diabetes: non insulin requiring autoimmune diabetes (NIRAD) 4. Diabetes Care.

[5] Mayr A, Schlosser M, Grober N (2007). GAD autoantibody affinity and epitope specificity identify distinct immunization profiles in children at risk for type 1 diabetes. Diabetes.

[6] Buzzetti R, Di Pietro S, Giaccari A (2007). High titer of autoantibodies to GAD identifies a specific phenotype of adult-onset autoimmune diabetes. Diabetes Care.

[7] Falorni A, Gambelunghe G, Forini F (2000). Autoantibody recognition of COOH-terminal epitopes of GAD65 marks the risk for insulin requirement in adult-onset diabetes mellitus. J Clin Endocrinol Metab.

[8] Krause S, Landherr U, Agardh CD (2014). GAD autoantibody affinity in adult patients with latent autoimmune diabetes, the study participants of a GAD65 vaccination trial. Diabetes Care.

[9] Williams AJ, Lampasona V, Schlosser M (2015). Detection of antibodies directed to the N-terminal region of GAD is dependent on assay format and contributes to differences in the specificity of GAD autoantibody assays for type 1 diabetes. Diabetes.

[10] Bonifacio E, Yu L, Williams AK (2010). Harmonization of glutamic acid decarboxylase and islet antigen-2 autoantibody assays for national institute of diabetes and digestive and kidney diseases consortia. J Clin Endocrinol Metab.

