# Supplementary material for: Autoantibodies to N-terminally truncated GAD improve clinical phenotyping of individuals with adult-onset diabetes: Action LADA 12
Source: Diabetologia. 2018 Apr 4;61(7):1644–9. doi: 10.1007/s00125-018-4605-3 (PMC6445455; doi:10.1007/s00125-018-4605-3)
Supplement: Supplementary file 1 — (PDF 89 kb) [file 125_2018_4605_MOESM1_ESM.pdf]

ESM Figure 1

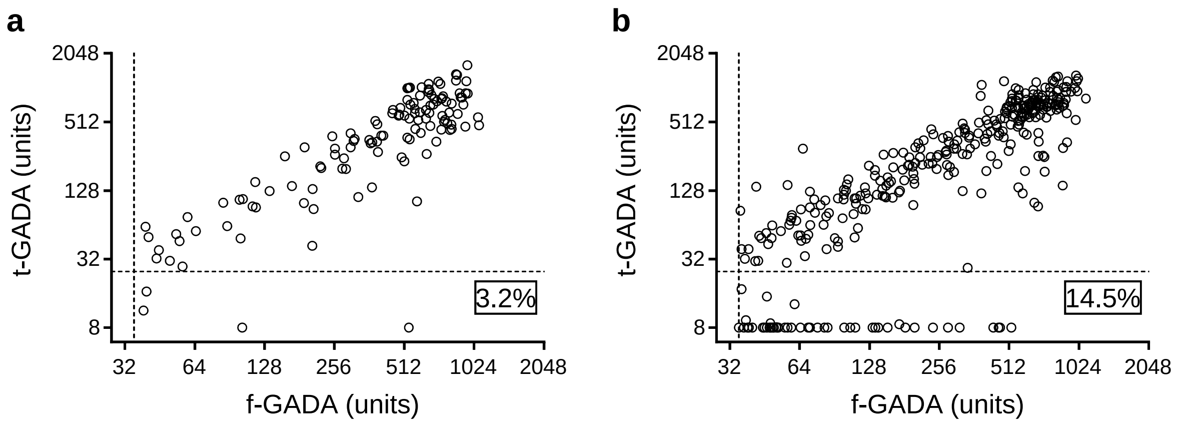

ESM Figure 1 Relationship between GADA epitope reactivity and beta cell autoantibody status. Levels of full-length GAD65 autoantibodies (f-GADA; x-axis) are plotted against levels of N-terminally truncated (aa 96-585) GAD65 autoantibodies (t-GADA; y-axis) using $\log_2$ scales for 126 f-GADA-positive individuals with adult-onset diabetes who also had IA-2A and/or ZnT8A (**a**) and 352 f-GADA-positive individuals with adult-onset diabetes who were negative for IA-2A and ZnT8A (**b**), respectively. Dashed lines indicate thresholds for positivity for f-GADA (35 units) and t-GADA (25 units). The frequencies of f-GADA-positive but t-GADA-negative individuals are 3.2% (4 of 126) in panel (**a**), versus 14.5% (51 of 352) in panel (**b**) (p=0.0003)
